# Evaluation of the effects of photodynamic therapy with phosphorus 31 magnetic resonance spectroscopy

**DOI:** 10.1038/sj.bjc.6690332

**Published:** 1999-04

**Authors:** M Nishiwaki, Y Fujise, T O Yoshida, E Matsuzawa, Y Nishiwaki

**Affiliations:** Departments of Microbiology and Immunology, Chemistry, Second Department of Internal Medicine, Hamamatsu University School of Medicine, 3600 Handa-cho Hamamatsu, 431-3192, Japan; Hamamatsu Photonics KK, 812 Joko-cho Hamamatsu, Japan; Hamamatsu Medical Center, 328 Tomitsuka-cho, Hamamatsu, 432, Japan

**Keywords:** photodynamic therapy (PDT), phosphorus 31 magnetic resonance spectroscopy (^31^P MRS), YAG-optical parametric oscillator laser (YAG-OPO laser), ATX-S10, HeLa cell tumour

## Abstract

Magnetic resonance spectroscopy in situ was used to study changes in phosphorus 31 metabolism after photodynamic therapy (PDT) of transplanted HeLa cell tumours. Tumours were irradiated 2 h after administration of ATX-S10 (8-formyloximethylidene-7-hydroxy-3-ethenyl-2,7,12,18, tetramethyl-porphyrin-13,17-bispropionil aspartate), a new photosensitizer and chlorin derivative. Nuclear magnetic resonance spectra were measured prior to illumination and 1, 3, 7, 14, 21 and 28 days after PDT on each mouse. A drastic decrease in adenosine triphosphate (ATP) and a concomitant increase in inorganic phosphate (Pi) were evident on the first day after PDT in all cases. The β-ATP/total phosphate (P) ratio was 0.64 ± 0.29% (average ± s.d.) in complete response, 0.67 ± 0.30% in recurrence and 2.45 ± 0.93% in partial response. Comparison of this ratio to the histological findings revealed that the β-ATP/total P ratio reflects the HeLa cell tumours which survived PDT. In other words, partial response on the one hand was distinguished from complete response and recurrence on the other with this ratio 1 day after PDT (*P* < 0.05). In addition, the ratio of phosphomonoester (PME) to Pi rose beyond 1.0 when macroscopic recurrence occurred, while it stayed under 1.0 in complete response. This finding suggests that the recurrence of HeLa cell tumours can be detected by the PME/Pi ratio. © 1999 Cancer Research Campaign


*British Journal of Cancer* (1999) **80**(1/2), 133–141
© 1999 Cancer Research Campaign
Article no. bjoc.1998.0332

### Evaluation of the effects of photodynamic therapy with phosphorus 31 magnetic resonance spectroscopy

**M Nishiwaki[1,3], Y Fujise[2], TO Yoshida[1,*], E Matsuzawa[4] and Y Nishiwaki[5]**

Departments of [1]Microbiology and Immunology, [2]Chemistry, [3]Second Department of Internal Medicine, Hamamatsu University School of Medicine, 3600 Handa-cho Hamamatsu 431-3192, Japan; [4]Hamamatsu Photonics KK, 812 Joko-cho Hamamatsu, Japan; [5]Hamamatsu Medical Center, 328 Tomitsuka-cho Hamamatsu 432, Japan

**Summary** Magnetic resonance spectroscopy in situ was used to study changes in phosphorus 31 metabolism after photodynamic therapy (PDT) of transplanted HeLa cell tumours. Tumours were irradiated 2 h after administration of ATX-S10 (8-formyloximethylidene-7-hydroxy-3-ethenyl-2,7,12,18, tetramethyl-porphyrin-13,17-bispropionil aspartate), a new photosensitizer and chlorin derivative. Nuclear magnetic resonance spectra were measured prior to illumination and 1, 3, 7, 14, 21 and 28 days after PDT on each mouse. A drastic decrease in adenosine triphosphate (ATP) and a concomitant increase in inorganic phosphate (Pi) were evident on the first day after PDT in all cases. The β-ATP/total phosphate (P) ratio was 0.64 ± 0.29% (average ± s.d.) in complete response, 0.67 ± 0.30% in recurrence and 2.45 ± 0.93% in partial response. Comparison of this ratio to the histological findings revealed that the β-ATP/total P ratio reflects the HeLa cell tumours which survived PDT. In other words, partial response on the one hand was distinguished from complete response and recurrence on the other with this ratio 1 day after PDT ($P < 0.05$). In addition, the ratio of phosphomonoester (PME) to Pi rose beyond 1.0 when macroscopic recurrence occurred, while it stayed under 1.0 in complete response. This finding suggests that the recurrence of HeLa cell tumours can be detected by the PME/Pi ratio.

**Keywords:** photodynamic therapy (PDT); phosphorus 31 magnetic resonance spectroscopy ($^{31}$P MRS); YAG-optical parametric oscillator laser (YAG-OPO laser); ATX-S10; HeLa cell tumour

Photodynamic therapy (PDT) is a cancer treatment modality based on the dye-sensitized photooxydation of biological matter in the target tissue which is now undergoing clinical trials in several countries (Foote et al, 1990; Henderson and Dougherty, 1992; Fisher et al, 1995; Kato, 1996). The actual agent mediating cytotoxicity appears to be singlet oxygen (Weishaupt et al, 1976). The mechanisms involved in PDT tumour destruction are not yet fully understood. Analysis of the metabolic changes occurring in the tumour cells in vivo in the host body is a useful method to evaluate the effects of PDT.

Phosphorus 31 magnetic resonance spectroscopy ($^{31}$P MRS) provides a non-invasive analytical method for monitoring levels of high-energy phosphorus-containing compounds. The use of a surface coil allows sequential measurement of a tumour in situ in the same animal. Several investigations have dealt with the metabolic changes in phosphorus compounds in tumours during and after PDT by using $^{31}$P MRS. It is well-known that a drastic decrease in adenosine triphosphate (ATP) and concomitant inorganic phosphate (Pi) elevation occurs in tumours shortly after PDT treatment with Photofrin II (Ph-II) (Ceckler et al, 1986; Naruse et al, 1986; Chopp et al, 1987; Hilf et al, 1987; Mattiello et al, 1990; Chapman et al, 1991). A similar phenomenon occurs after PDT with disulphonated phthalocyanine (AlS2Pc) (Bremner et al, 1994) and also with δ-aminolaevulinic acid (ALA) (Hua et al, 1995). Moreover, Bremner et al (1994) have shown in their MRS studies on real-time changes during and after PDT that this Pi elevation starts within 15 min after PDT. Chapman et al (1991) have shown that the ratio of the concentration of Pi to that of β-ATP increases when the tumour hypoxic fraction increases after PDT. These phenomena coincide with the in vitro studies of Hilf et al in which they reported that Ph-II-induced PDT inhibits the mitochondrial enzymes (Gibson and Hilf, 1983; Hilf et al, 1984; Perlin et al, 1985) and the plasma membrane enzymes (Gibson et al, 1988) leading to reduction in cellular ATP levels and loss of viability (Hilf et al, 1986) in R3230AC mammary adenocarcinomas. Moreover, Chopp et al have reported that the early metabolic response of mammary carcinoma to PDT is laser dose-dependent with sequential measurement of $^{31}$P MRS up to 7 days after PDT (Chopp et al, 1990). The heterogeneous metabolic response which induces the heterogeneous tumour response to PDT has been shown using in vivo localized $^{31}$P MRS (Ceckler et al, 1991). These studies suggest it is possibile to non-invasively predict the biological outcome to treatment in the early period after PDT by the metabolic response of the tumour. These hypotheses, however, have not been tested with long-term observations after PDT.

The results of this study support the hypothesis that it is possible to predict the biological outcome by the early metabolic response of HeLa cell tumours after PDT with sequential measurements of

*Received 28 May 1998*
*Revised 4 October 1998*
*Accepted 21 October 1998*

*Correspondence to:* Masako Nishiwaki, The Second Department of Internal Medicine, Hamamatsu University School of Medicine, 3600 Handa-cho Hamamatsu 431-3192 Japan

*Present address: Department of Microbiology and Immunology, Showa University School of Medicine, 1-8-5 Hatanodai Shinagawa, Tokyo 142, Japan

NMR spectra over a period of up to 28 days. ATX-S10, a new photosensitizer and chlorine derivative developed to decrease phototoxicity, was used in this study. Moreover, we also show that tumour recurrence can be detected by $^{31}P$ MRS monitoring.

### MATERIALS AND METHODS

### Animals and tumour line

Female BALB/c nude mice were supplied by Japan SLC Inc. (Hamamatsu, Japan), and housed four per each cage and kept under specific pathogen-free conditions. The mice were 8 weeks old and weighed 18–21 g when the experiment started. HeLa cells (strain; 15S3D) of $5 \times 10^6$ 0.1 ml$^{-1}$ were implanted intracutaneously into the right side of the back. The tumours were measured in three orthogonal diameters (D1, D2 and D3) at 2- to 3-day intervals with a caliper before and after PDT. PDT treatment was given 9–12 days after inoculation when the tumour was hemispherical in shape and had reached the desired size, approximately 10–12 mm in D1, 7–9 mm in D2 and 4–6 mm in D3. No spontaneous necrosis was observed at these sizes.

### Chemicals

The photosensitizing drug ATX-S10 was synthesized, purified and provided by Toyo Hakka Kogyo Co. Ltd (Okayama, Japan). The chemical structure is shown in Figure 1. This compound is water-soluble and features rapid body clearance. The absorption maximum is at 670 nm (Takemura et al, 1993). The solution of ATX-S10 (2 mg ml$^{-1}$) for injection was made by dissolving phosphate-buffered saline (PBS) at a pH of 7.4, and 25 mg kg$^{-1}$ was injected intraperitoneally 2 h before laser irradiation. PBS was locally prepared by dissolving 8 g sodium chloride, 0.2 g potassium chloride, 1.15 g of sodium hydrogenphosphate ($Na_2HPO_4$) and 0.2 g of potassium dihydrogenphosphate ($KH_2PO_4$) to 1 liter of aqueous solution and adjusting its pH to 7.4.

### Fluorescence detection of ATX-S10 in the tumour

The accumulation of ATX-S10 in the tumours was detected by fluorescence using photonic multichannel analyser 10 (PMA 10; Hamamatsu Photonics KK, Hamamatsu, Japan). The tumours were excited at 410 nm, and the fluorescence emission was scanned from 300 to 800 nm, resulting in the appearance of a distinct peak positioned at 670 nm.

### PDT efficiency

An yttrium aluminum garnet-optical parametric oscillator laser (YAG-OPO laser; Hamamatsu Photonics KK, Hamamatsu, Japan) was used to generate light at a wavelength of 670 nm. The beam was transmitted through a 800 µm fibre. The output energy was 5 mJ per pulse, the repetition rate was 15 Hz, the pulse width was 5 n's and the power density was 75 mW. Laser irradiation was performed from 13 directions around the tumour at a distance of 3–5 mm from the tumour surface. The duration of light exposure was 1333 s, for a total light dose of 100 J per tumour. Mice with the appropriate tumours were divided into four groups: group 1 (four mice), given neither ATX-S10 nor light exposure; group 2 (four mice), irradiated without ATX-S10 administration; group 3 (five mice), given ATX-S10 without light exposure; group 4 (20 mice), irradiated 50 or 100 J per tumour with ATX-S10.

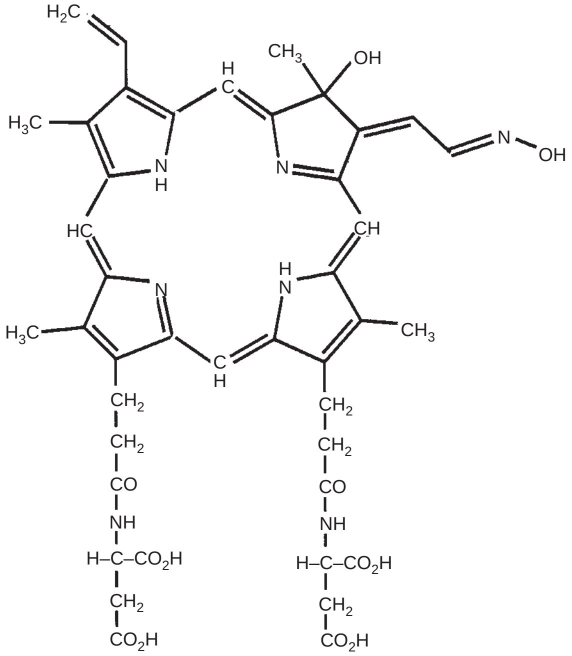

**Figure 1** Chemical structure of ATX S10

### NMR spectroscopy

NMR spectra were obtained at 109.25 MHz for $^{31}P$ on a JEOL-GSX 270 WB spectrometer (JEOL, Tokyo, Japan) with a 9 cm bore 6.4 Tesla vertical-type magnet. The magnetic field homogeneity was optimized by observing the $^{1}H$ signal from the water in the tumour. $^{31}P$ MRS was obtained using a pulse width of 15 µs, corresponding to the optimal signal. Each spectrum allowed for 512 acquisitions collected. Typical acquisition parameters for the $^{31}P$ NMR spectra were as follows: 10 KHz spectral width, 2048 data point, 0.1 second acquisition time and 1.6 second repetition time. The data were collected as a set of 13.7 min.

### Phosphorus metabolism

A home-built hemispherical three-turn surface coil, with the largest turn having a major axis of 13 mm and a minor axis of 9 mm to fit the initial tumour size, was positioned over the HeLa cell tumour. The surface coil used in this study has a high resolution and a height/half-width ratio exceeding 100, with the signal contribution from normal tissue adjacent to the tumour minimized by its hemispherical shape. Therefore, spectra from larger tumours contained very little contribution from underlying muscle, while it was taken into consideration that those from smaller tumours may have contained a contribution from non-tumour tissue. For the NMR study, the mice were anaesthetized using an intraperitoneal injection of 10% PBS solution containing 50 mg kg$^{-1}$ of pentobarbital, which produced an anaesthetized state for about 60 min. The mice were taped to the NMR probe, and this probe was set in the magnet bore vertically. The measurement of $^{31}P$NMR spectra was done just before PDT, and 1, 3, 7, 14, 21 and 28 days after PDT. A small bottle including 25 µl of 1 mol hexamethyl phosphoric triamide (HMPA) solution was attached to the centre of the surface coil as an external reference. We adopted this reference halfway

© Cancer Research Campaign 1999

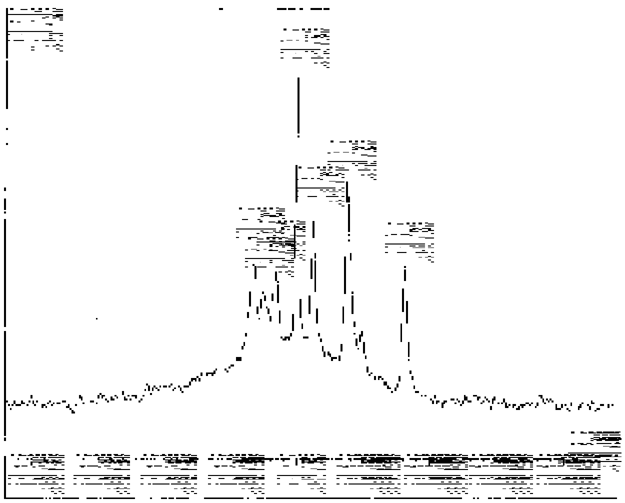

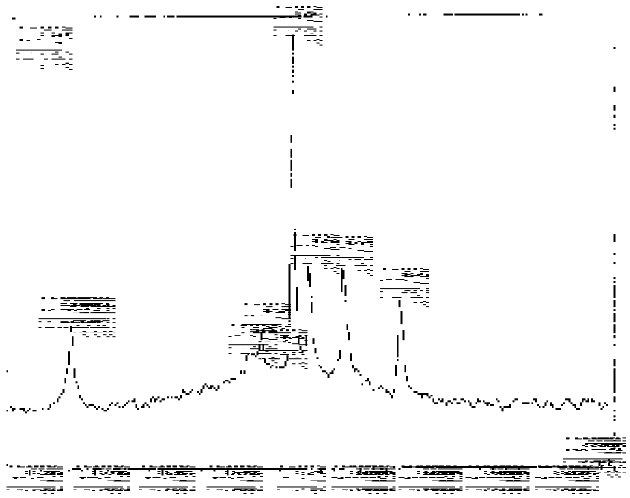

**Figure 2** (**A**) ³¹P spectrum of progressing HeLa tumour. There are seven sharp peaks. From left to right: 1, phosphomonoester (PME); 2, inorganic phosphate (Pi); 3, phosphodiester (PDE); 4, phosphocreatine (PCr); 5, γ-ATP; 6, α-ATP; 7, β-ATP. (**B**) ³¹P spectrum obtained from a surface coil placed over the back without tumour. Peak assignments correspond to those described above through this study to confirm the index of the high resolution. Therefore, the HMPA peak is not found in every ³¹P NMR spectrum chart. The spectra were analysed as follows. The area under each peak was calculated by approximating each peak to a triangular shape after extrapolation to the baseline. Total P is the sum of the areas of seven sharp peaks and the broad ³¹P component which was generated under these sharp peaks. The β-ATP/Pi ratio and β-ATP/total P ratio, values which represent the metabolic status of the tissue, were calculated and compared with the various biological outcomes. For statistical analysis, a Student's *t*-test was used to obtain a comparison of each treatment outcome. For all tests, a two-sided *P*-value of less than 0.05 was considered to be a statistically significant difference.

### Histological study

Confirmation of the biological outcome: in the standard experiment, just after the 28-day MRS measurement, the whole tumour tissue, including underlying muscle, was removed to evaluate the PDT effect histopathologically. Comparison of ³¹P MRS with the histopathological findings: next, in order to compare ³¹P MRS with the histopathological findings, we additionally prepared eight tumours in the same fashion as group 4, and excised them on the first day, when the biological outcome was suspected with ³¹P MRS after PDT with 50–100 J laser irradiation. Detection of the tumour recurrence: in yet another experiment, when tumour recurrence was predicted by ³¹P MRS, the small nodules were excised to certify the recurrence histologically in three cases. The samples fixed in 20% formaldehyde solution were dehydrated and embedded in paraffin. Sections were cut and routinely stained with haematoxylin and eosin.

### RESULTS

### ³¹P NMR spectrum of HeLa cell tumours

A representative spectrum of HeLa cell tumours 9–13 days after transplantation is illustrated in Figure 2(A). The tumour spectrum is characterized by high levels of phosphocreatine (PCr), ATP and phosphomonoester (PME). In particular, the PME/Pi ratio exceeded 1.0 in tumours 10 mm in diameter. In experiments not presented, the natural course of untreated HeLa cell tumours was investigated with the multiple sampling of spectra 3–40 days after transplantation. The spectrum obtained 8–19 days after transplantation showed the same spectrum pattern. A spectrum obtained from the 13 × 9 mm surface coil positioned on the back of mice without tumours in order to know the background of the tumour is shown in Figure 2(B). A large PCr peak with low level of PME peaking is present, which is different from that of HeLa cell tumours.

### Biological outcome

In the present study, biological outcome after PDT was divided into three categories. In four cases the tumours disappeared and no regrowth appeared until 28 days after PDT (complete response, CR), in eight cases the tumours disappeared by the 7th day, but regrew after the 10th day (recurrence, RECUR) and the eight other cases showed only tumour reduction (partial response, PR). This biological outcome was histopathologically confirmed. Tumours irradiated without ATX-S10 administration (group 2) and tumours given ATX-S10 without light exposure (group 3) showed no differences with the no treatment control (group 1) in tumour size or colour. The macroscopic findings of the tumours shortly after PDT (group 4) were as follows: RECUR showed almost the same macroscopic change as CR within 7 days after PDT. On the 1st day after PDT, no differences in tumour size among three categories (PR, RECUR and CR) were noted. On the 3rd day, reduction in tumour size in every PR was evident and remarkable shrinkage was observed in every CR and RECUR. On the 7th day crusts were formed in CR and RECUR and the once reduced tumour regrew in PR.

### Evaluation of the PDT effect

#### *Evaluation in the early period after PDT with ³¹P NMR*

Figure 3 shows the representative pre- and day 1 spectra of the results. In the cases of CR and RECUR, a striking increase in Pi, a

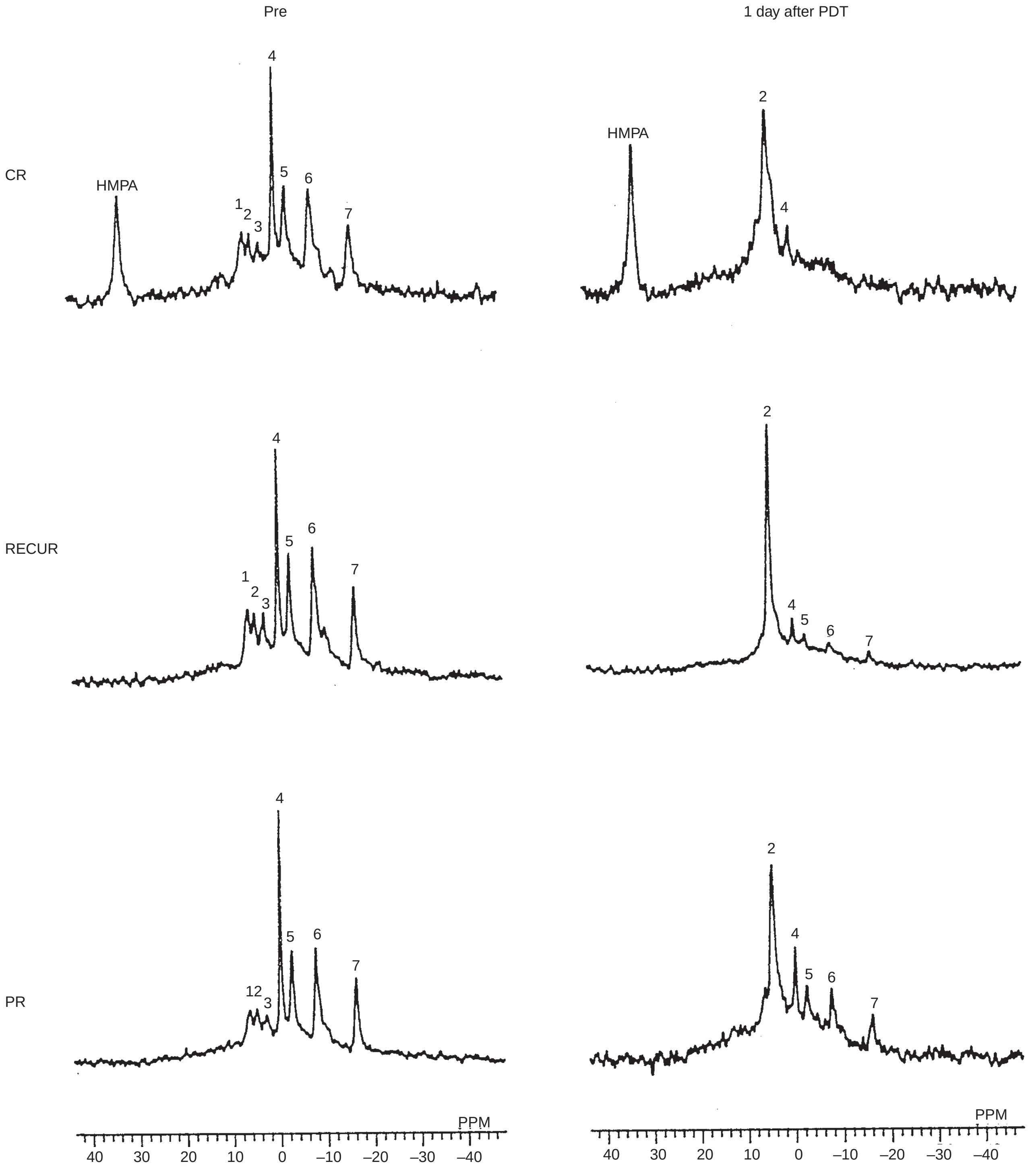

**Figure 3** Representative $^{31}P$ spectra of respective biological outcome measured pretreatment and 1 day after PDT. Peak assignments correspond to those described in Figure 2

drastic decrease in ATP, and a decrease in PME, phosphodiester (PDE) and PCr were observed on the 1st day after PDT. In addition, there was a broad $^{31}P$ component, presumably from cell debris, upon which the cell spectrum is superimposed. A considerable amount of ATP was residual in PR, on the one hand, while the ATP had almost disappeared in CR and RECUR, on the other hand, in the day 1 spectrum. The PR pattern is definitely different from that of CR and RECUR. In order to quantify this phenomenon, the β-ATP/Pi ratio which is used as a useful metabolic indicator is plotted against the time after PDT up to the 7th day (Figure 4). There is no difference among the values of the untreated control, the group given ATX-S10 only and the group that under-

*© Cancer Research Campaign 1999*

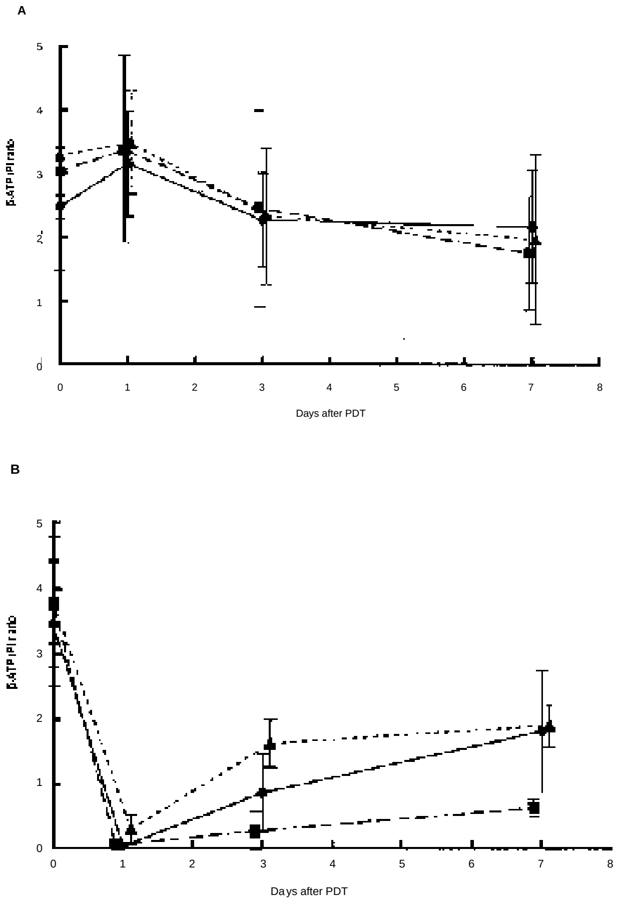

**Figure 4** Changes in $^{31}$P metabolism occurring after PDT up to 7 days are shown by plotting the β-ATP/Pi ratio for each group; data for the untreated control (◆), ATX-S10 only (■) and laser only (▲) groups are also shown (**A**). CR (■), RECUR (◆), PR (▲) (**B**). Bars (s.d.) are based on between four and eight individual animals

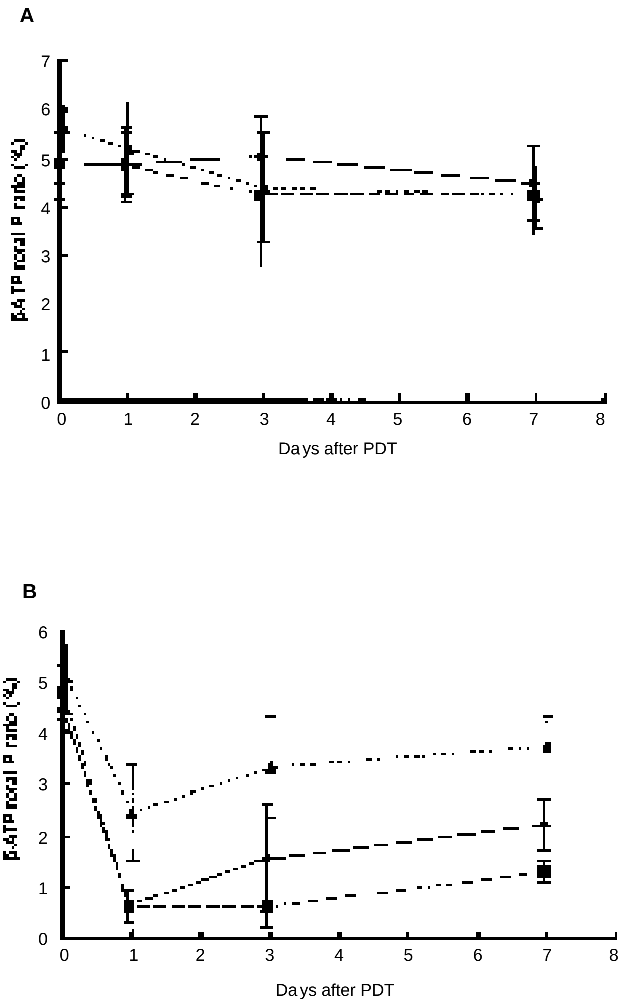

**Figure 5** Showing the effect on $^{31}$P metabolism by plotting the β-ATP/total P ratio for each group: data for the untreated control (◆), ATX-S10 (■) only and laser only (▲) groups are also shown (**A**). CR (■), RECUR (◆), PR (▲) (**B**). Bars (s.d.) are based on between four and eight individual animals went laser irradiation only. A significant decrease in the 1st day value is observed for the PDT group in which the tumours of CR and RECUR indicate a greater decrease than that of PR ($P$ = 0.0170 by the Student's $t$-test comparing CR vs PR, $P$ = 0.0115 by the Student's $t$-test comparing RECUR vs PR). This ratio remains at a low value up to the 7th day in the CR group. On the contrary, in the case of RECUR, this ratio returns to the same value as PR by the 7th day after treatment ($P$ = 0.0093 by the Student's $t$-test comparing RECUR vs CR). Next, the ratio of β-ATP to total P was plotted against the time after PDT up to the 7th day to analyse the metabolic change based on ATP hydrolysis quantitatively (Figure 5). One day after PDT, the β-ATP/total P ratio was 0.64 ± 0.29% (average ± s.d.) in CR, 0.67 ± 0.30% in RECUR and 2.45 ± 0.93% in PR, which enabled us to distinguish PR from RECUR and CR ($P$ = 0.0008 by the Student's $t$-test comparing RECUR vs PR and $P$ = 0.0007 CR vs PR). There was no difference between RECUR and CR in this ratio ($P$ = 0.8504 by the Student's $t$-test).

### *Comparison of $^{31}$P MRS and pathological evaluation*

Figures 6 and 7 show the $^{31}$P MRS and micrograph of a highly effective case which displayed the same pattern as CR and RECUR 1 day after PDT, and one which displayed the same pattern as PR, respectively. These results indicate that PR is distinguished from CR and RECUR by the $^{31}$P MRS 1 day after PDT.

### Detection of recurrence

The representative time progression of spectra obtained from pretreatment up to 28 days after PDT is shown in Figures 8 and 9. Figure 8 shows the sequential measurement in the case of CR in which no re-increase in PME and PDE was detected up to 56 days after PDT. Contrastingly, in the case of RECUR, a re-increase in PME and PDE, which had maintained low levels while the tumour was microscopically undetectable, was observed when the tumour was detected macroscopically (Figure 9). PME/Pi ratio exceeded 1.0 in the progressive HeLa cell tumour as shown in Figure 1. We investigated the correlation between recurrence and PME/Pi ratio by calculation of all CR and RECUR cases (Table 1). The value of this ratio in every CR case remains under 1.0, while in every RECUR case this ratio rises above 1.0 when macroscopic

© Cancer Research Campaign 1999

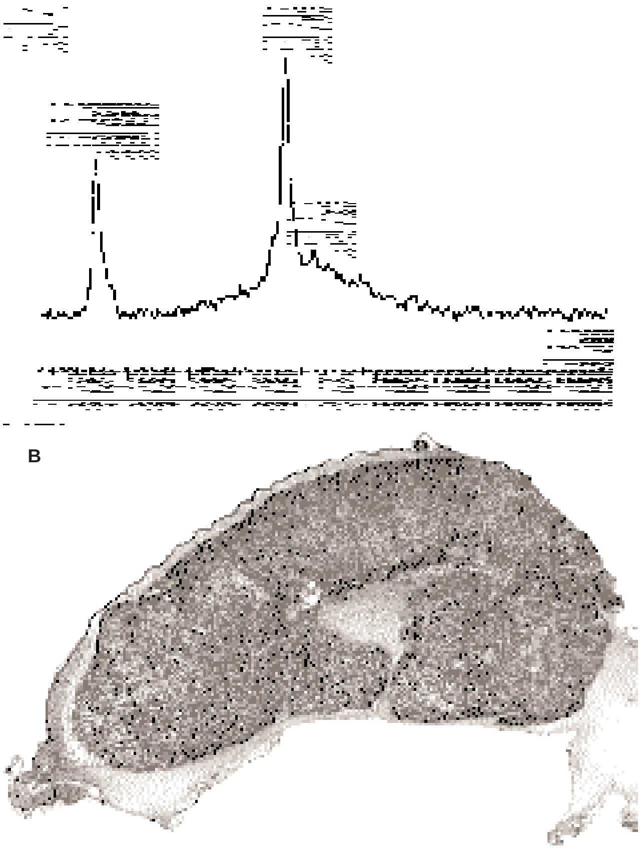

**Figure 6** Representative $^{31}$P spectrum (**A**) and micrograph (**B**) of highly effective case on the 1st day after PDT. The β-ATP/total P ratio of this MRS is 0.002. Live HeLa cells were not observed in (**B**). Peak assignments in (**A**) correspond to those described in Figure 2. (**B**), Original magnification × 3.3

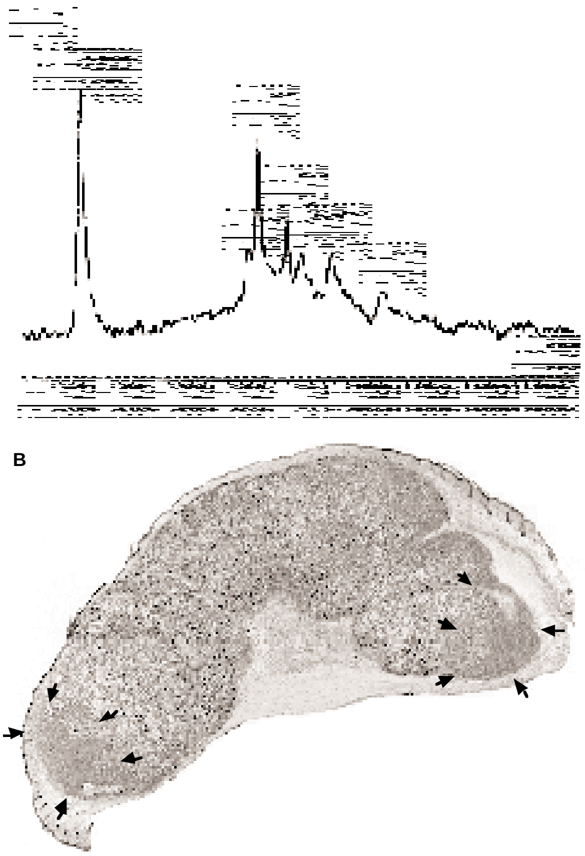

**Figure 7** Representative $^{31}$P spectrum (**A**) and micrograph (**B**) of PR on the 1st day after PDT. The β-ATP/total P ratio of this MRS is 0.016. Live HeLa cells were observed (arrow) in (**B**). Peak assignments in (**A**) correspond to those described in Figure 2. (**B**) Original magnification × 3.3

regrowth is detected. A follow-up study up to the 28th day after PDT might make it possible to predict recurrence according to the changing value of this ratio. Regrowing HeLa cell tumours were detected in all three cases that were histologically studied when the tumour recurrence was suspected by the $^{31}$P MRS. Figure 10 shows one of these cases.

### DISCUSSION

Recent developments in NMR spectroscopy have shown convincingly that it represents a versatile technique for the examination of several important aspects of tumour metabolic changes, not only in untreated growth but also in various therapeutic responses (Evanochko et al, 1982; Ng et al, 1982; Daly et al, 1987; Daly and Cohen, 1989; Steen, 1989; Dewhirst et al, 1990; Negendank, 1992). Recognition of the initial metabolic change after PDT may be very useful in order to understand the mechanisms of tumour cell death based on the PDT treatment. In addition, in this study, we succeeded in not only observing this initial response with ATX-S10-PDT, but also in observing the changing process of HeLa cell tumour metabolism in the four CR, the eight RECUR and the eight PR cases by a follow-up study of $^{31}$PMRS up to 28 days after PDT.

We adopted a new photosensitizer, ATX-S10, which is a water-soluble chlorine derivative. It has been reported that ATX-S10 has the highest retention between 4 and 6 h, and clears rapidly after 24 h, after intravenous injection (Nakajima et al, 1993). We measured fluorescence in the tumour using the PMA 10 prior to this research. Peak concentration was detected 2–4 h after intraperitoneal injection and almost diminished 24 h after injection (unpublished data). We irradiated ‘2 h’ after administration of ATX-S10 based on this study. Hashimoto and Sakata reported that the PDT with 25 mg kg$^{-1}$ administration of ATX-S10 and 125J cm$^{-2}$ laser irradiation of human oesophageal squamous cell carcinoma transplanted in subcutaneous tissue of nude mice had a significant effect in observations up to 7 days after treatment (Hashimoto and Sakata, 1994). Therefore, we chose a dose of 25 mg kg$^{-1}$ ATX-S10 for administration and 100 J per tumour laser irradiation. To investigate the appropriate laser dose prior to this study, tumours were irradiated with 50, 75 and 100 J. Irradiation with 50 J resulted in PR in all cases. Various results were recognized in both the 75- and 100-J groups, with no differences between these two groups. Therefore, we chose 50 and 100 J irradiation. We considered that the varying biological outcome in the 100-J group was due to the heterogeneous response of the tumour against PDT, composed of the concentration and distribution of photosensitizing drug in the irradiated tissue, the incident photon flux delivered to the tissue and the tissue oxygen concentration, as shown by Ceckler et al (1991). ATX-S10–PDT with these doses allowed us to obtain various metabolic patterns based on the treatment outcome. We selected these doses as increasing the efficacy was not the aim of this study. In order to increase the

*© Cancer Research Campaign 1999*

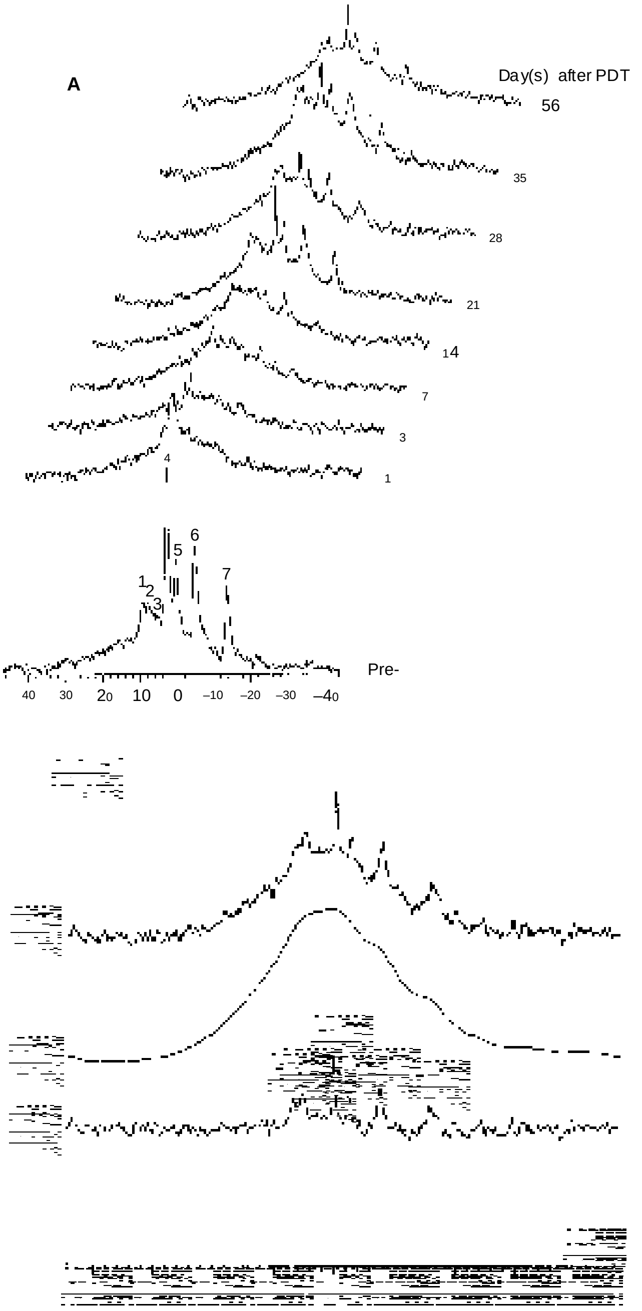

Figure 8 (A) Multiple measurement of $^{31}$P spectra of a HeLa cell tumour resulted in complete response (CR) after PDT. There was no tumour regrowth detected until 161 days after PDT. A drastic decrease of ATPs was observed on the 1st day and re-increase of PME and PDE was never detected. Spectral intensities have been scaled relative to the first spectrum. Peak assignments correspond to those described in Figure 2. (**B**) *a*, Original day 28 spectrum, 512 data accumulations with a 30-Hz line broadening exponential multiplication; *b*, same as *a* except a 400-Hz line broadening exponential multiplication was used; *c*, difference spectrum of *a* minus *b*. This spectrum resembled that of the normal muscle (Figure 2B)

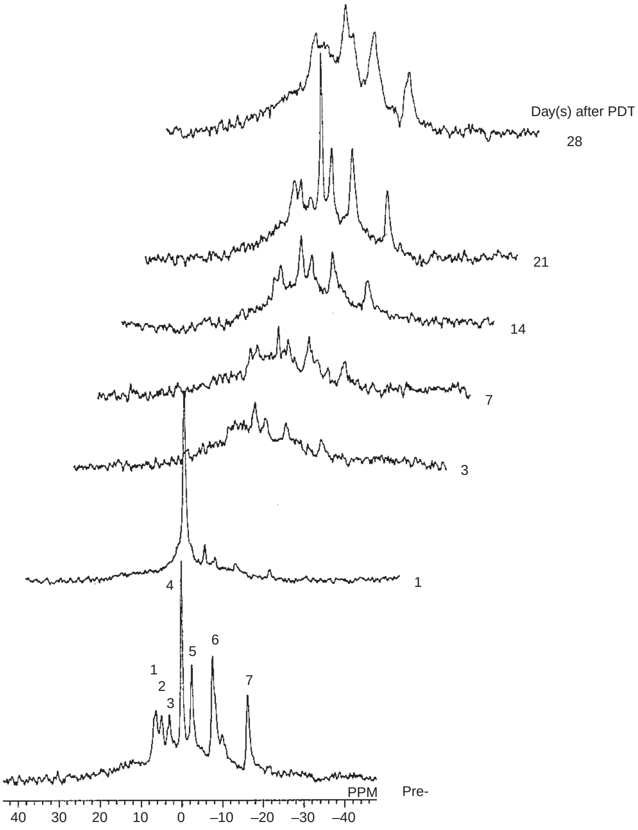

Figure 9 Multiple measurement of $^{31}$P spectra of a HeLa tumour resulted in recurrence (RECUR) after PDT. The day 1 spectrum is similar to that of CR. The regrowth of small tumours (5 mm in diameter) was macroscopically detected on the 20th day. The day 21 spectrum shows the progressing HeLa cell tumour pattern: increased PME and PDE. Spectral intensities have been scaled relative to the first spectrum. Peak assignments correspond to those described in Figure 2

efficacy, more investigation about photosensitizer dose, laser dose and the interval between photosensitizer administration and laser irradiation are needed. We obtained the following important findings from this study.

First, a drastic decrease in ATP and a concomitant increase in Pi occurred shortly after PDT with ATX-S10. This finding was similar to that of Ph-II–PDT (Ceckler et al, 1986; Naruse et al ,1986; Chopp et al, 1987; Hilf et al, 1987; Mattiello et al, 1990; Chapman et al, 1991), AIS2Pc–PDT (Bremner et al, 1994) and ALA–PDT (Hua et al, 1995), and this result indicates tumour tissue hypoxia after treatment, which is supported by the morphological finding that vessel occlusions have developed in many vessels within 1 day after ATX-S10–PDT (Hayashi et al, 1998).

Secondly, the detailed quantitative analysis of phosphorus metabolites makes it possible to classify the effect of PDT. The Pi-dominant pattern lasted for 3 days in all cases, with a decrease amplitude thereafter, as was the case with the Naruse report (Naruse et al, 1986). An ATP decrease lasted for 3 days as well. In the PR case, ATP never disappeared, contrastingly in the RECUR case, ATP peaks began to reappear at 3 days. In the CR case they began to reappear after 7 days (Figure 8), when the underlying intact region was detected because of the shrinkage of the tumour. Thus, the ATP peaks that appear after 7 days in the case of CR are contributed to the non-tumour region as shown in Figure 8B; the spectral patterns of CR have changed to the muscle pattern.

**Table 1** The detection of recurrence after PDT treatment based on the PME/Pi ratio

| Mouse No. | Pre- | Day 1 | Day 3 | Day 7 | Day 14 | Day 21 | Day 28 | Result | Tumour reappearing |
|---|---|---|---|---|---|---|---|---|---|
| G4-3 | 1.61 | 0.09 | 0.52 | 0.97 | 0.78 | 1.08[a] | 1.14 | RECUR | 20 |
| G4-4 | 1.14 | 0.18 | 0.55 | 0.79 | 0.91 | 0.77 | 1.65[a] | RECUR | 17 |
| G5-1 | 3.12 | 0.03 | 0.28 | 0.84 | 3.08[a] | 1.52 | –[d] | RECUR | 10 |
| G5-3[b] | 3.33 | 0.05 | 0.15 | 0.50 | 1.38[a] | 1.54 | 1.73 | RECUR | 21 |
| G5-4 | 2.48 | 0.08 | 0.70 | 0.70 | 0.70 | 2.27[a] | 1.69 | RECUR | 21 |
| G11-2 | 1.97 | 0.18 | 0.18 | 0.88 | 1.60[a] | 1.78 | –[d] | RECUR | 10 |
| G11-4 | 1.88 | 0.07 | 0.04 | 0.65 | 1.39[a] | 1.05 | –[e] | RECUR | 21 |
| G13-3 | 2.28 | 0.14 | 0.77 | 0.75 | 1.36[a] | 2.30 | 2.15 | RECUR | 10 |
| G3-2[c] | 1.82 | 0.13 | 0.76 | 0.38 | 0.98 | 0.45 | –[f] | CR | |
| G3-3 | 1.21 | 0.18 | 0.53 | 0.12 | 0.81 | 0.80 | 0.65 | CR | |
| G11-3 | 1.78 | 0.07 | 0.26 | 0.38 | 0.80 | 0.78 | –[e] | CR | |
| G18-2[b] | 2.02 | 0.08 | 0.14 | 0.54 | 0.90 | 0.67 | 0.84 | CR | |

[a]PME/Pi ratio beyond 1.0 was observed first after PDT. [b]ATX-S10 30 mg $kg^{-1}$ was administered. [c]Laser 75 J per tumour was irradiated. [d]This mouse was sacrificed on the 21st day for a pathological study. [e]This mouse died of unknown causes on the 21st day. [f]This mouse died of unknown causes on the 24th day. CR = complete response; RECUR = recurrence.

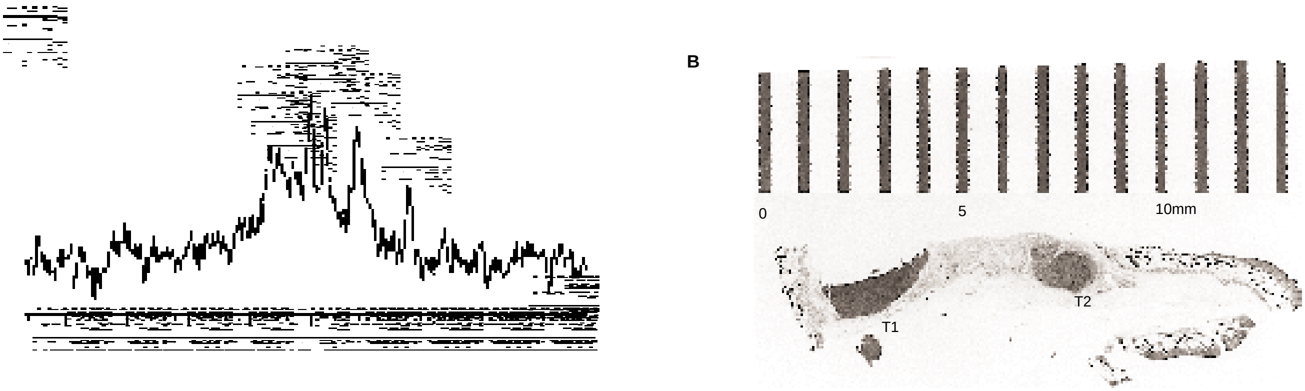

**Figure 10** Representative $^{31}P$ spectrum (**A**) and the micrograph (**B**) when recurrence was suspected. In this case, tumour regrowth was macroscopically observed on the 14th day after treatment. On that day, a small nodule was excised just after $^{31}P$ MRS measurement. The PME/Pi ratio is 1.17 (beyond 1.0), and the small HeLa cell tumours of 3 mm in diameter (T1) and of 1.5 mm in diameter (T2) were observed in the specimen. T2 was not observed macroscopically before tumour excision. The surface coil was put on the area including both these tumours. Peak assignments in (**A**) correspond to those described in Figure 2. (**B**) Original magnification × 3.3

Although we did not refer to the change in absolute value of the metabolites in this study, the change in absolute value of ATP will give us an important indication to evaluate the PDT effect. We are intending to investigate the absolute value of the metabolites in the near future by comparing the area of each peak to the area of the HMPA, which we adopted in this study as an external reference.

Additionally, quantitative analysis of ATP was performed. We obtained results in this study that agree with several researchers who adopted the β-ATP/Pi ratio as a therapeutic index. Next we focused on the ratio of the β-ATP concentration to the total phosphate. In this ATX-S10–PDT it was revealed that β-ATP/total P ratio needs to decrease under 1% 1 day after PDT in order to expect CR, as is shown in Figure 5. This means that PR is distinguished from RECUR and CR by the β-ATP/total P ratio. The β-ATP/total P ratio is more sensitive than the β-ATP/Pi ratio for evaluating the ATX-S10–PDT effect the next day. Next, $^{31}P$ MRS 1 day after PDT, in which the PDT effect was predictable, was compared with the tumour specimen. Laser doses of 50 and 100 J were adopted intentionally in order to produce partial response cases and highly effective cases representing CR or RECUR cases ($n$ = 8). In the case of PR, the residue ATP on the 1st day was shown to be derived from the surviving HeLa cell, compared with highly effective cases in which no live HeLa cells were observed and ATP was diminished (Figures 6 and 7). This result proved the β-ATP/total P ratio which indicates that the ATP concentration to the total P in the surface coil reflects the live cell volume in the treated HeLa cell tumour. As mentioned above, PR was distinguished from RECUR and CR on the 1st day by the β-ATP/total P ratio, while there was no difference between RECUR and CR in this ratio. As to the distinction between RECUR and CR, the β-ATP/Pi ratio may possibly be one parameter. It became clear that the time course response of β-ATP/Pi ratio reflects the therapeutic 'dose' of PDT, as Chopp suggested (Chopp et al, 1990). Moreover, in the present study, we found that this ratio remained low for up to 7 days in the CR cases. Contrastingly, this ratio in the RECUR cases reverted to the same level as that in the PR cases within 7 days after PDT (Figure 4). By using this parameter, RECUR may possibly be distinguished from CR within 7 days after treatment.

The last important finding in this study was about the detection of recurrence. It became clear, by the sequential measurement of $^{31}P$ MRS up to 28 days after PDT, that the PME/Pi ratio rose beyond 1.0 when recurrence occurred. As is shown in Table 1, this ratio of G5-3 and G11-4 exceeded 1.0 prior to macroscopic detection of regrowth. This shows that it is possible to predict HeLa cell tumour recurrence using the PME/Pi ratio. This was based on the fact that the PME/Pi ratio was beyond 1.0 in viable HeLa cell tumours and that this ratio was under 1.0 in the background tissue. In all specimens excised when recurrence was suspected based on the PME/Pi ratio, tumour regrowth was evident (Figure 10). The case shown in Figure 10 was the smallest recurrent tumour that was diagnosed in this study by $^{31}P$ MRS. The detection of cancer within normal tissue using the characteristics of cancers in $^{31}P$ MRS has recently been studied (Ng et al, 1982; Evanochko et al, 1982; Negendank, 1992). If the prominent metabolic characteristics of various tumours are detected, it will be possible to predict the recurrence of those tumours by detecting the characteristics of $^{31}P$ MRS like these HeLa cell tumours used in this study.

In conclusion, $^{31}P$ MRS is a non-invasive and very useful method of evaluating the effects of PDT and investigating its mechanisms.

### ACKNOWLEDGEMENTS

The authors acknowledge Mr T Matsuo (Department of Microbiology, Hamamatsu University School of Medicine) for his continued assistance in the maintenance of HeLa cells and Mr T Hirano (Hamamatsu Photonics KK) for his advice about laser irradiation. We thank Dr I Sakata (Toyo Hakka Kogyo Co. Ltd) for the valuable sample of ATX-S10. We are also grateful to Emeritus Professor T Yoshimi, Professor H Nakamura and Dr T Kawasaki (Second Department of Internal Medicine, Hamamatsu University School of Medicine) for their support and advice on this work. We would also like to thank Professor M Unger (Pennsylvania Hospital, Philadelphia) for much discussion and criticism of this manuscript.

### REFERENCES

Bremner JCM, Bradley JK, Stratford IJ and Adams GE (1994) Magnetic resonance spectroscopic studies on ‘real-time’ changes in RIF-1 tumor metabolism and blood flow during and after photodynamic therapy. *Br J Cancer* **69**: 1083–1087

Ceckler TL, Bryant RG, Penny DP, Cibson SL and Hilf R (1986) $^{31}P$ NMR spectroscopy demonstrates decreased ATP levels in vivo as an early response to photodynamic therapy. *Biochem Biophys Res Commun* **140**: 273–279

Ceckler TL, Gigson SL, Kennedy SD, Hilf R and Bryant RG (1991) Heterogeneous tumor response to photodynamic therapy assessed by in vivo localised $^{31}P$ NMR spectroscopy. *Br J Cancer* **63**: 916–922

Chapman JD, McPhee MS, Walz N, Chemer MP, Stobbe CC, Soderlind K, Arnfield M, Meeker BE, Trimble L and Allen PS (1991) Nuclear magnetic resonance spectroscopy and sensitizer-adduct measurements of photodynamic therapy-induced ischemia in solid tumors. *J Natl Cancer Inst* **83**: 1650–1659

Chopp M, Farmer H, Hetzel F and Schaap AP (1987) In vivo $^{31}P$ NMR spectroscopy of mammary carcinoma subjected to subcurative photodynamic therapy. *Photochem Photobiol* **46**: 819–822

Chopp M, Hetzel FW and Jiang Q (1990) Dose-dependent metabolic response of mammary carcinoma to photodynamic therapy. *Radiation Res* **121**: 288–294

Daly PF and Cohen JS (1989) Magnetic resonance spectroscopy of tumors and potential in vivo clinical application: a review. *Cancer Res* **49**: 770–779

Daly PF, Lyon RC, Faustino PJ and Cohen JS (1987) Phospholipid metabolism in cancer cells monitored by $^{31}P$ NMR spectroscopy. *J Biolog Chem* **262**: 14875–14878

Dewhirst MW, Sostman HD, Leopold KA, Charles HC, Moor D, Burn RA, Tucker JA, Harrelson JM and Oleson JR (1990) Soft-tissue sarcomas; MR imaging and MR spectroscopy for prognosis and therapy monitoring. *Radiology* **174**: 847–853

Evanochko WT, Ng TC, Glickson JD, Durant JR and Corbett TH (1982) Human tumors as examined by in vivo $^{31}P$ NMR in athymic mice. *Biochem Biophs Res Commun* **109**: 1346–1352

Fisher AMR, Murphree AL and Gomer CJ (1995) Clinical and preclinical photodynamic therapy. *Lasers Surg Med* **17**: 2–31

Foote CS (1990) Chemical mechanisms of photodynamic action. In *Proc. SPIE Institute on Advanced Optical Technologies on Photodynamic Therapy*. *SPIE* **6**: 115–126

Gibson SL and Hilf R (1983) Photosensitization of mitochondrial cytochrome c oxidase by hematoporphyrin derivative and related porphyrins in vitro and in vivo. *Cancer Res* **43**: 4191–4197

Gibson SL, Murant RS and Hilf R (1988) Photosensitizing effect of hematoporphyrine derivative and photofrin II on the plasma membrane enzymes 5´-nucleotidase, $Na^+$ $K^+$ -ATPase, and $Mg^{2+}$ -ATP ase in R3230 AC mammary adenocarcinomas. *Cancer Res* **48**: 3360–3366

Hashimoto Y and Sakata I (1994) Photodynamic therapy for transplantable human esophageal cancer (ES4) of nude mice with excimer dye laser and ATX-S10. *J Jpn Society Laser Med* **15**: 1–7

Hayashi Y, Yoshida TO, Nishiwaki M, Matsuzawa E, Yicheng L and Ohta I (1998) Effective mechanisms of ATX-S10, a new photosensitizer, against HeLa tumors in nude mice. *Jpn J Chemother* (in press)

Henderson BW and Dougherty TJ (1992) How does photodynamic therapy work? *Photochem Photobiol* **55**: 145–157

Hilf R, Smail DB, Murant RS, Leakey PB and Gibson SL (1984) Hematoporphyrine derivative-induced photosensitivity of mitochondrial succinate dehydrogenase and selected cytosolic enzymes of R3230AC mammary adenocarcinomas of rat. *Cancer Res* **44**: 1483–1488

Hilf R, Murant RS, Narayanan U and Gibson SL (1986) Relationship of mitochondrial function and cellular adenosine triphosphate levels to hematoporphyrin derivative-induced photosensitization in R3230AC mammary tumors. *Cancer Res* **46**: 211–217

Hilf R, Penney DP, Ceckler TL and Bryant RG (1987) Early biochemical responses to photodynamic therapy monitored by NMR spectroscopy. *Photochem Photobiol* **46**: 809–817

Hua Z, Gibson SL, Foster TH and Hilf R (1995) Effectiveness of δ-aminolevulinic acid-induced protoporphyrin as a photosensitizer for photodynamic therapy in vivo. *Cancer Res* **55**: 1723–1731

Kato H (1996) History of photodynamic therapy: past, present, and future. *Jpn J Chemother* **23**: 8–15

Mattiello J, Evelhoch L, Brown E, Schaap AP and Hetzel FW (1990) Effect of photodynamic therapy on RIF-1 tumor metabolism and blood flow examined by $^{31}P$ and $^{2}H$ NMR spectroscopy. *NMR Biomed* **3**: 64–70

Nakajima S, Hayashi H, Sakata I and Takemura T (1993) New photosensitizer-ATX-S10-having type 1 photoreaction. *Igaku no Ayumi* **164**: 187–188

Naruse S, Horikawa Y, Tanaka C, Higashi T, Sekimoto H, Ueda S and Hirakawa K (1986) Evaluation of the effects of photoradiation therapy on brain tumors with in vivo $^{31}P$ MR spectroscopy. *Radiology* **160**: 827–830

Negendank W (1992) Studies of human tumors by MRS: a review. *NMR Biomed* **5**: 303–324

Ng TC, Evanochko WT, Hiramoto RN, Ghanta VK, Durant JR and Glickson JD (1982) $^{31}P$ NMR spectroscopy of in vivo tumors. *J Mag Res* **49**: 271–286

Perlin DS, Murant RS, Gibson SL and Hilf R (1985) Effects of photosensitization by hematoporphyrin derivative on mitochondrial adenosine triphosphate-mediated proton transport and membrane integrity of R3230AC mammary adenocarcinomas. *Cancer Res* **45**: 653–658

Steen RG (1989) Response of solid tumors to chemotherapy monitored by in vivo $^{31}P$ nuclear magnetic resonance spectroscopy: a review. *Cancer Res* **49**: 4075–4085

Takemura T, Nakajima S and Sakata I (1993) Photophysicochemical properties of photosensitizer ATX-70, ATX-S10 and fluorescent diagnostic agent HAT-D01. *J Jpn Society Laser Med* **14**: 21–29

Weishaupt KR, Gomer CJ and Dougherty TJ (1976) Identification of singlet oxygen as the cytotoxic agent in photo-inactivation of murine tumor. *Cancer Res* **36**: 2326–2329

© Cancer Research Campaign 1999
